# Development of a hemoptysis risk prediction model for patients following CT-guided transthoracic lung biopsy

**DOI:** 10.1186/s12890-020-01282-9

**Published:** 2020-09-16

**Authors:** Saibin Wang, Ke Dong, Wei Chen

**Affiliations:** 1 Department of Respiratory Medicine, Jinhua Municipal Central Hospital, No. 365, East Renmin Road, Jinhua, 321000 Zhejiang Province China; 2 Department of Nuclear Medicine, Jinhua Municipal Central Hospital, No. 365, East Renmin Road, Jinhua, 321000 Zhejiang Province China; 3 Department of Radiology, the Second Affiliated Hospital and Yuying Children’s Hospital of Wenzhou Medical University, Wenzhou, 325027 Zhejiang Province China

**Keywords:** Lung, Biopsy, Hemoptysis, Nomogram

## Abstract

**Background:**

Computed tomography-guided transthoracic needle biopsy (CT-TNB) is a widely used method for diagnosis of lung diseases; however, CT-TNB-induced bleeding is usually unexpected and this complication can be life-threatening. The aim of this study was to develop and validate a predictive model for hemoptysis following CT-TNB.

**Methods:**

A total of 436 consecutive patients who underwent CT-TNB from June 2016 to December 2017 at a tertiary hospital in China were divided into derivation (*n* = 307) and validation (*n* = 129) cohorts. We used LASSO regression to reduce the data dimension, select variables and determine which predictors were entered into the model. Multivariate logistic regression was used to develop the predictive model. The discrimination capacity of the model was evaluated by the area under the receiver operating characteristic curve (AUROC), the calibration curve was used to test the goodness-of-fit of the model, and decision curve analysis was conducted to assess its clinical utility.

**Results:**

Five predictive factors (diagnosis of the lesion, lesion characteristics, lesion diameter, procedure time, and puncture distance) selected by LASSO regression analysis were applied to construct the predictive model. The AUC was 0.850 (95% confidence interval [CI], 0.808–0.893) in the derivation, and 0.767 (95% CI, 0.684–0.851) in the validation. The model showed good calibration consistency (*p* > 0.05). Moreover, decision curve analysis indicated its clinical usefulness.

**Conclusion:**

We established a predictive model that incorporates lesion features and puncture parameters, which may facilitate the individualized preoperative prediction of hemoptysis following CT-TNB.

## Background

Currently, low-dose computed tomography (CT) is recommended for lung cancer screening in routine clinical practice at many certified medical centers [1, 2]. This recommendation has contributed to increment the number of CT-detectable pulmonary lesions, including asymptomatic pulmonary nodules and masses [3]. Generally, in the presence of lesions larger than 10 mm or smaller lesions with a rapid growth rate, additional diagnostic procedures would be needed, such as transthoracic lung biopsy guided by CT, transbronchial lung biopsy guided by endobronchial ultrasound or virtual bronchoscopic navigation, or surgery [1, 4]. Among these procedures, CT-guided transthoracic needle biopsy (CT-TNB) is usually the preferred method because it is minimally invasive, has higher diagnostic accuracy and a lower cost [4–6]. However CT-TNB also has complications, such as hemoptysis, which occurs with a frequency of 0.5–14.4%, and intrapulmonary hemorrhage, with an even higher rate of 2.9–54.5% [4, 5].

Nowadays, CT-TNB is routinely performed on an outpatient basis in many medical units. In this scenario, an important issue concerning outpatient management is not the occurrence of hemoptysis per se, but hemoptysis requiring hemostatic therapy and patient hospitalization. Therefore, it would be helpful if clinicians could predict the risk of post-CT-TNB hemoptysis in clinical practice. Unfortunately, to our knowledge, there is no recommended model for the prediction of post-CT-TNB hemoptysis.

In the present study, we retrospectively investigated patients’ clinical characteristics, lesion features, and CT-TNB parameters, in order to establish a predictive model based on valuable predictors of post-CT-TNB hemoptysis.

## Methods

### Participants

A total of 436 consecutive patients who underwent CT-TNB at a tertiary hospital (Jinhua Hospital of Zhejiang University, Jinhua, China) from June 2016 to December 2017 were enrolled in this study. Of these, 309 patients were randomly assigned to the derivation dataset, while the remaining patients (*n* = 127) were assigned to the validation cohort. This study was performed in accordance with the Declaration of Helsinki, which was revised in 1983. The study was approved by the ethics committee of Jinhua Hospital of Zhejiang University (No. 2018001008). All patient information was handled anonymously and informed consent was therefore waived.

### Variables collection

Preoperative laboratory examinations, lesion features, and surgical procedure information were extracted from hospital information system [7]. Clinical characteristics included gender, age, heart rate, systolic and diastolic blood pressure, room-air oxygen saturation, and coexisting chronic obstructive pulmonary disease (yes/no). Laboratory examinations included prothrombin time, platelets, D-dimer, serum tumor markers (carcinoembryonic antigen, squamous cell carcinoma antigen, and CYFRA21-1), fasting blood glucose, triglyceride, and C-reactive protein. Lesion features included the diagnosis of the lesion, lesion diameter (1–2 cm, 2–3 cm, and ≥ 3 cm), lesion location I (left/right lung), lesion location II (hilum, upper/lower lung, or middle lung), lesion characteristics (ground-glass, solid, or cavitary lesion), lesion burr (yes/no), the shortest distance from the lesion to nearby vessel (< 10 mm, or ≥ 10 mm), CT-attenuation value. Surgical procedure information included puncture position (supine, prone, or lateral position), procedure time, puncture distance (the straight-line distance from the pleura to the center of the lesion), biopsy times, and hemoptysis after procedure (yes/no) [7].

### CT-TNB procedure

Participants received CT-TNB in different positions on the basis of the calculation of the shortest distance from the center of the lesion to the body surface. All biopsies were performed by a qualified physician, who has been performed more than 5000 cases of CT-TNB in the past 20 years. In this study, a coaxial 18-gauge needle (Lot Number, REXK0682; Bard Peripheral Vascular, Inc., Tempe, AZ) was used for all biopsies [7]. Generally, two biopsies were performed; however, additional biopsies were required when needed [7].

Patients were requested to maintain the supine position for at least 6 h following CT-TNB and were allowed to get out of bed 24 h later. In this study, we defined ≥1 new post-CT-TNB bloody sputum episode as hemoptysis (new meaning that the patient had never had bloody sputum or hemoptysis before the biopsy). Generally, patients with limited hemoptysis spontaneously regained hemostasis, and treatment was only required when the severity of hemoptysis increased. The patient who had post-CT-TNB severe hemoptysis would be required to rest in bed absolutely, and be encouraged to cough up blood clots. Hemocoagulase and vasopressin were used for hemostasis.

### Statistical analysis

Multiple imputation method was applied to account for missing data. Summary statistics were used to describe subject characteristics. Continuous data were expressed as medians (interquartile range), and categorical data were presented as the number and the percentage. The unpaired *t*-test or the Mann-Whitney U test, the Pearson chi-squared test or the Fisher’s exact test was used to compare characteristics between the derivation and validation cohorts. In this study, we followed the methods as previously described [7]. The least absolute shrinkage and selection operator (LASSO) regression method was applied to filter potential predictors, and logistic regression analysis was used to develop the predictive model for post-CT-TNB hemoptysis. Nomogram was constructed to facilitate the use in clinical practice. The discrimination ability of the model was assessed by calculating the area under the receiver operating characteristic curve (AUROC). Calibration curve was constructed and the unreliability test was performed to evaluate the goodness-of-fit of the model. The model was assessed for clinical utility using decision curve analysis (DCA) [8]. R software (version 3.5.1; https://www.r-project.org) was used for statistical analyses, and statistical significance was defined as a *p*-value < 0.05.

## Results

In the derivation cohort, 43.7% (134/307, 95% confidence interval [CI], 38.1–49.2%) of patients experienced hemoptysis following CT-TNB, and 2 (1.5% of the hemoptysis) cases required hemostasis treatment. In the validation cohort, the incidence of hemoptysis was 41.1% (53/129, 95% CI, 32.6–49.6%), and 1 (1.9% of the hemoptysis) patient required hemostasis treatment. One patient died from severe postoperative blood loss. This patient was a 53-year-old man with hypertension and uremia. A solid mass with a diameter of 3.3 cm in the right middle lobe was found on CT scan, and the shortest distance from the center of the lesion to the body surface was 1.5 cm. This patient received CT-TNB in supine position and only one biopsy was performed. The postoperative pathological result of the lesion was pulmonary inflammatory pseudotumor. Patient’s clinical characteristics, laboratory tests, and biopsy parameters are shown in Table 1.

**Table 1:** Patient’s clinical characteristics, laboratory tests, and biopsy parameters

Variables	Overall cohort	Derivation cohort	Validation cohort	***P***-value
	No (***n*** = 436)	Yes (***n*** = 307)	(*n* = 129)	
Hemoptysis, n (%)	187 (42.89)	134 (43.65)	53 (41.09)	0.622
Gender, n (%)				0.848
Female	166 (38.07)	116 (37.79)	50 (38.76)	
Man	270 (61.93)	191 (62.21)	79 (61.24)	
Age, (year)	63 (54–70)	63 (54–71)	63 (54–70)	0.836
SBP, (mmHg)	128 (117–141)	128 (117–142)	130 (116–139)	0.933
DBP, (mmHg)	78 (70–84)	78 (70–84)	76 (70–83)	0.322
HR, (beat/min)	80 (72–88)	80 (72–87)	80 (68–89)	0.814
SPO_2_, (%)	98 (96–98)	97 (96–98)	98 (96–99)	0.040
COPD, n (%)				0.429
No	309 (70.87)	221 (71.99)	88 (68.22)	
Yes	127 (29.13)	86 (28.01)	41 (31.78)	
PT, (s)	12.40 (11.50–13.30)	12.40 (11.50–13.25)	12.40 (11.70–13.40)	0.475
D-Dimer, (mg/mL)	591.0 (320.0–1269.8)	611.9 (337.0–1361.0)	544.0 (290.0–988.0)	0.501
Platelets, (× 10^9^/L)	231 (177–285)	235 (179–286)	226 (171–285)	0.493
CRP, (mg/L)	3.95 (0.80–24.92)	4.50 (0.90–25.00)	3.00 (0.60–24.80)	0.519
Blood glucose, (mmol/L)	5.38 (4.74–6.71)	5.37 (4.75–6.69)	5.39 (4.74–6.77)	0.419
Triglyceride, (mmol/L)	1.12 (0.86–1.52)	1.11 (0.84–1.54)	1.14 (0.90–1.42)	0.556
SCC, (μg/L)	0.70 (0.50–1.20)	0.70 (0.50–1.05)	0.80 (0.60–1.30)	0.132
CYFRA21-1, (ng/mL)	2.55 (1.50–5.26)	2.40 (1.45–4.95)	2.80 (1.60–6.40)	0.094
CEA, (ng/mL)	2.20 (1.11–6.85)	2.21 (1.08–7.90)	2.17 (1.26–6.35)	0.455
Lesion diagnosis, n (%)				0.887
Benign	187 (42.89)	131 (42.67)	56 (43.41)	
Malignant	249 (57.11)	176 (57.33)	73 (56.59)	
Lesion characteristic, n (%)				0.968
Ground-glass	15 (3.44)	11 (3.58)	4 (3.10)	
Solid	334 (76.61)	235 (76.55)	99 (76.74)	
Cavitary	87 (19.95)	61 (19.87)	26 (20.16)	
Location I, n (%)				0.661
Left lung	216 (49.54)	150 (48.86)	66 (51.16)	
Right lung	220 (50.46)	157 (51.14)	63 (48.84)	
Location II, n (%)				0.855
Hilus of lung	21 (4.82)	15 (4.89)	6 (4.65)	
Upper lung	220 (50.46)	155 (50.49)	65 (50.39)	
Middle lung	19 (4.36)	15 (4.89)	4 (3.10)	
Lower lung	176 (40.37)	122 (39.74)	54 (41.86)	
Lesion burr, n (%)				0.776
No	258 (59.17)	183 (59.61)	75 (58.14)	
Yes	178 (40.83)	124 (40.39)	54 (41.86)	
Distance from the closest vessel, n (%)				0.573
< 10 mm	214 (49.08)	148 (48.21)	66 (51.16)	
≥ 10 mm	222 (50.92)	159 (51.79)	63 (48.84)	
CT-attenuation value, (HU)	35.0 (28.0–43.0)	36.0 (28.5–43.0)	34.0 (27.0–42.0)	0.065
Lesion diameter, n (%)				0.752
1–2 cm	55 (12.61)	38 (12.38)	17 (13.18)	
2–3 cm	105 (24.08)	77 (25.08)	28 (21.71)	
≥ 3 cm	276 (63.30)	192 (62.54)	84 (65.12)	
Puncture distance, (mm)	10.0 (0.0–20.0)	10.0 (0.0–20.0)	10.0 (0.0–20.0)	0.737
Procedure time, (min)	10.0 (8.0–12.0)	9.0 (8.0–12.0)	10.0 (8.0–11.0)	0.684
Puncture times, median (IQR)	1 (1–1)	1 (1–2)	1 (1–1)	0.563
Biopsy times, median (IQR)	2 (2–2)	2 (2–2)	2 (2–2)	0.105
Puncture position, n (%)				0.347
Supine	153 (35.09)	113 (36.81)	40 (31.01)	
Prone	210 (48.17)	141 (45.93)	69 (53.49)	
Lateral position	73 (16.74)	53 (17.26)	20 (15.50)	

Footnotes: *SBP* systolic blood pressure, *DBP* diastolic blood pressure, *HR* heart rate, *SPO*_*2*_ pulse oximetry saturation, *COPD* chronic obstructive pulmonary disease, *CRP* C-reactive protein, *PT* prothrombin time, *SCC* squamous cell carcinoma antigen, *CEA* carcinoembryonic antigen, *NA* not applicable

**Fig. 1:**
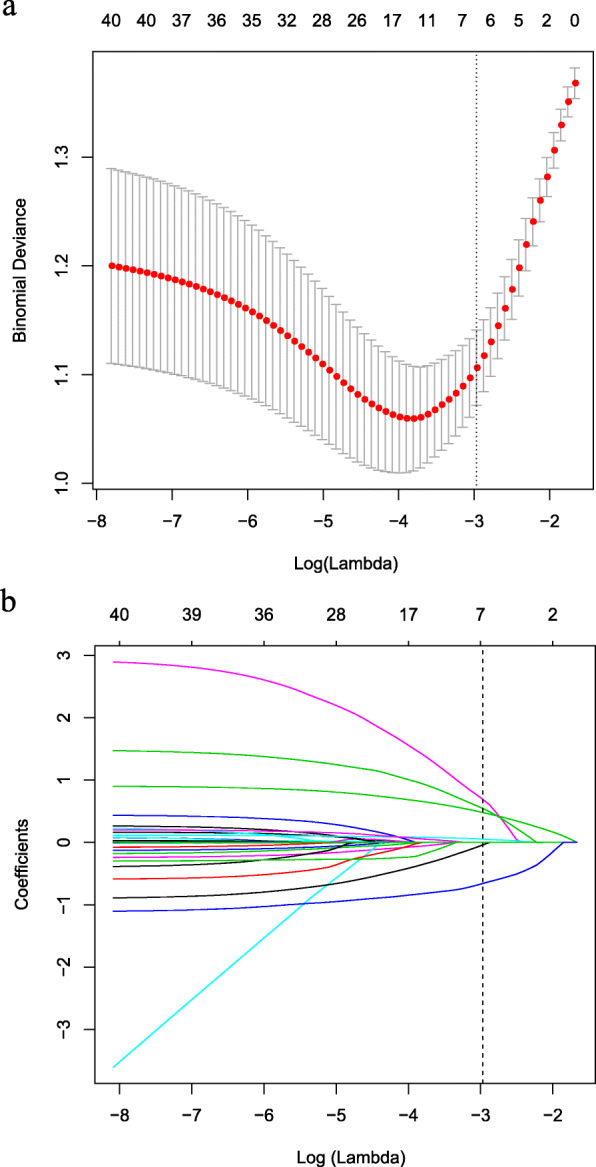
Predictors selection using the LASSO regression method with 10-fold cross-validation. Binomial deviance was plotted versus log (lambda) (**a**), and coefficients plots were produced against the log (lambda) sequence (**b**). A total of 29 variables were included in the LASSO regression method with 10-fold cross-validation. Dotted vertical lines in figures (**a**, **b**) were drawn at the optimal values by utilizing the 1-SE criteria, where five nonzero coefficients were filtered, and the corresponding five variables were lesion diagnosis, lesion characteristics, lesion diameter, procedure time, and puncture distance. These five variables were included in the predictive model. Footnotes: *LASSO* Least absolute shrinkage and selection operator, *SE* standard error

After analyzing the 309 patients in the derivation cohort, 29 variables were reduced to 5 potential predictors based on nonzero coefficients in the LASSO regression analysis (Fig. 1). These variables were: lesion diagnosis, lesion characteristics, lesion diameter, procedure time, and puncture distance.

**Fig. 2:**
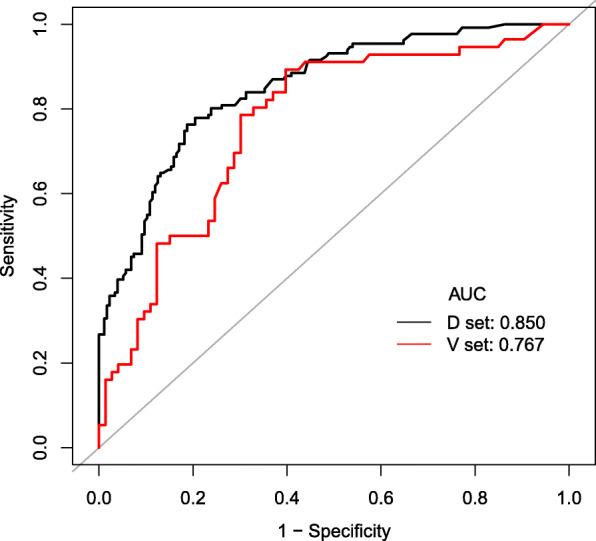
ROC curves of the predictive model in the derivation and validation datasets. The area under the ROC curve (black line) shows the predictive ability in the derivation cohort, and area under the ROC curve (red line) validates the predictive ability of the model. Footnotes: *ROC* receiver operating characteristic

We established a risk prediction model for CT-TNB-induced hemoptysis based on the aforementioned 5 predictors, which independently associated with the risk of post-CT-TNB hemoptysis as assessed by logistic regression analysis (Table 2). As shown in Fig. 2, the AUC for the predictive model (black line) was 0.850 (95% CI, 0.808–0.893), while the AUC for the validation (red line) was 0.767 (95% CI, 0.684–0.851). The optimal cut-off value of ROC curve was 0.45 based on the maximum principle of Youden index, where it yielded an accuracy of 79.2%, a sensitivity of 76.3%, a specificity of 81.3%, a positive predictive value of 75.2%, and a negative predictive value of 82.2%.

**Table 2:** Logistic regression analysis of each individual variable for the risk of hemoptysis following CT-TNB

Variables	Odds ratio	95% CI	*P* Value
Puncture distance	2.375	1.882–2.997	0.000
Lesion characteristic			
Ground-glass	Ref.		
Solid	0.037	0.004–0.360	0.004
Cavitary	0.081	0.008–0.830	0.034
Lesion diagnosis			
Benign	Ref.		
Malignant	0.270	0.145–0.500	0.000
Lesion diameter, (cm)			
1–2	Ref.		
2–3	1.154	0.472–2.823	0.754
≥ 3	0.361	0.148–0.879	0.025
Procedure time	1.111	1.006–1.228	0.038
Gender			
Female	Ref.		
Man	0.754	0.450–1.264	0.284
Age	1.012	0.989–1.035	0.314
SBP	0.990	0.973–1.007	0.252
DBP	1.008	0.982–1.036	0.550
HR	0.991	0.971–1.010	0.342
SPO_2_	1.044	0.883–1.234	0.651
COPD			
No	Ref.		
Yes	1.008	0.540–1.883	0.980
Location I			
Left lung	Ref.		
Right lung	1.208	0.737–1.977	0.454
Location II			
Hilus of lung	Ref.		
Upper lung	0.772	0.237–2.508	0.666
Middle lung	0.670	0.137–3.282	0.621
Lower lung	0.446	0.129–1.539	0.201
Lesion burr			
No	Ref.		
Yes	0.827	0.452–1.516	0.539
Distance from the closest vessel			
< 10 mm			
≥ 10 mm	0.972	0.545–1.734	0.925
CT-attenuation value	1.002	0.986–1.018	0.798
Puncture times			
< 3	Ref.		
≥ 3	3.048	0.590–15.757	0.184
Biopsy times	0.853	0.456–1.598	0.620
Puncture position			
Supine			
Prone	0.686	0.317–1.485	0.339
Lateral position	1.502	0.789–2.859	0.215

Footnotes: *CT-TNB* computed tomography-guided transthoracic needle biopsy, *SBP* systolic blood pressure, *DBP* diastolic blood pressure, HR heart rate, *SPO2* pulse oximetry saturation, *COPD* chronic obstructive pulmonary disease

**Fig. 3:**
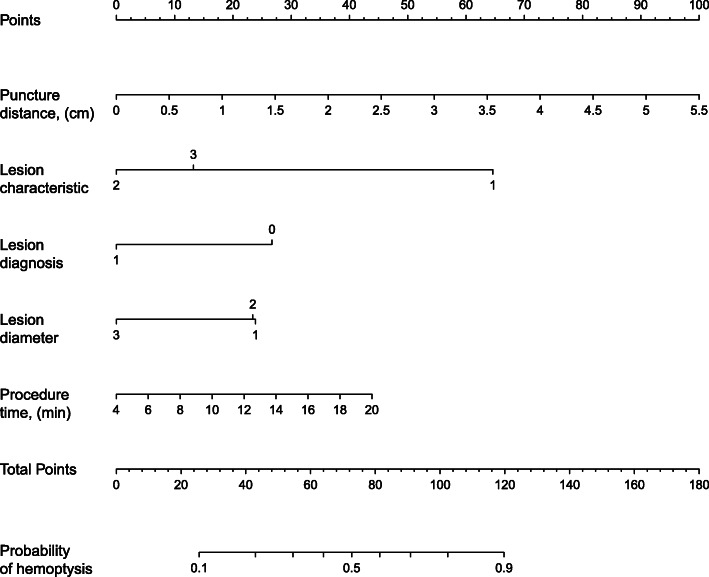
Nomogram for estimation of post-CT-TNB hemoptysis risk and its predictive performance. Lesion characteristic: 1: ground-glass, 2: solid, 3: cavitary; Diagnosis: 0: benign, 1: malignant; Lesion diameter: 1: 1-2 cm, 2: 2-3 cm, 3: ≥3 cm. Footnotes: *CT-TNB* computed tomography-guided transthoracic needle biopsy

**Fig. 4:**
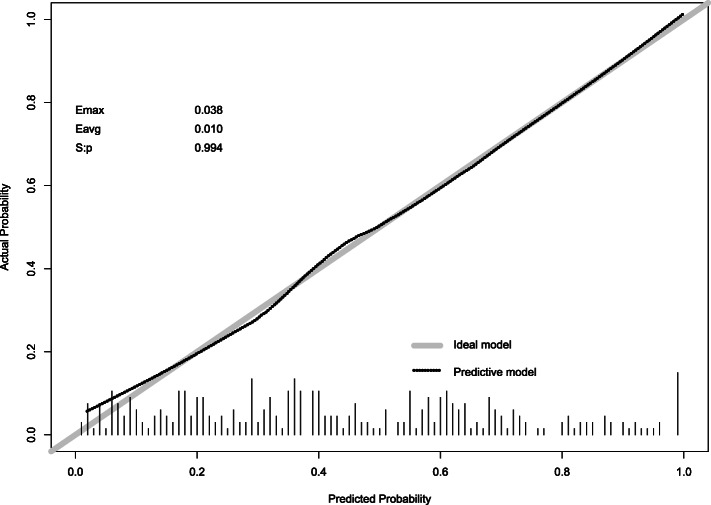
Calibration curve of the predictive model. The Y-axis represents the actual post-CT-TNB hemoptysis rate. The X-axis represents the predicted risk of post-CT-TNB hemoptysis. The gray diagonal line represents the ideal model, which means that the predicted value is completely consistent with the actual observation result. The dotted line represents the prediction model established in this study. A closer fit to the gray diagonal line represents a better prediction. In the unreliability test, it yielded a p-vale of 0.994, a Emax value of 0.038, and a Eavg value of 0.010, which indicates that there was no departure from a perfect fit of the calibration between our model and the ideal model. Footnotes: *CT-TNB* computed tomography-guided transthoracic needle biopsy

To provide clinicians with a quantitative tool to predict the risk of post-CT-TNB hemoptysis, a nomogram was constructed based on the predictive model (Fig. 3). The calibration curve of the model for the risk of post-CT-TNB hemoptysis shows good consistency between prediction and observation in the derivation cohort. The unreliability test yielded a *p*-value of 0.994, with a Emax of 0.038 and a Eavg of 0.010, which indicated that there was no departure from a perfect fit (Fig. 4).

**Fig. 5:**
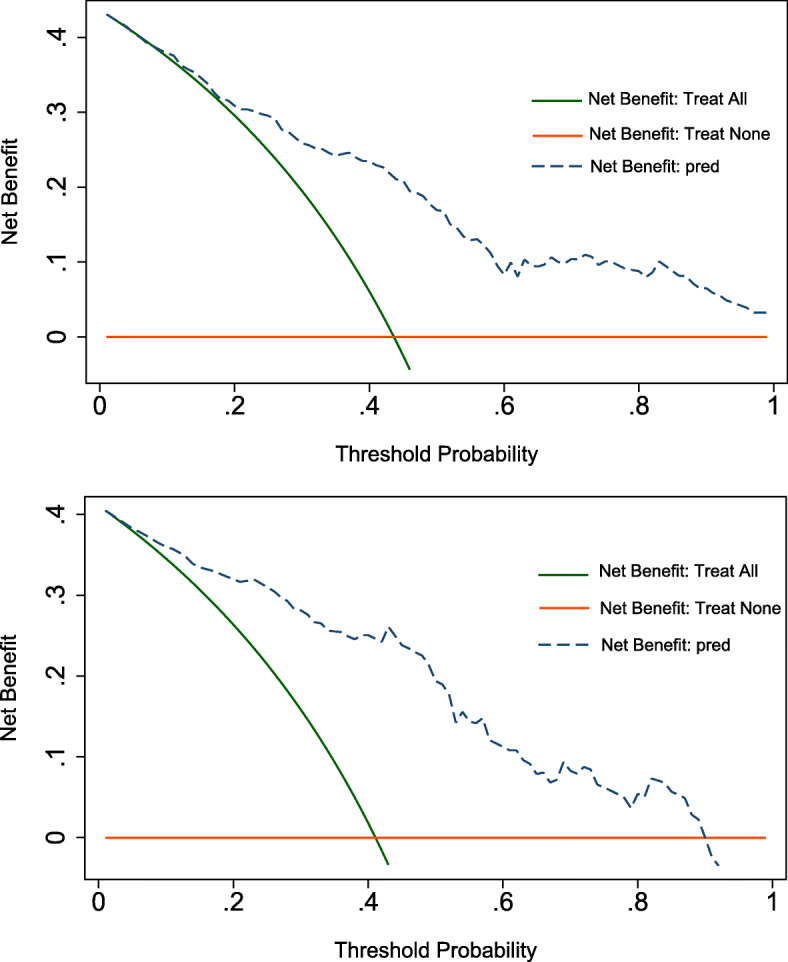
Decision curve analysis for the predictive model and the validation. The Y-axis measures the net benefit. The dashed line represents the model. The black line represents the assumption that all patients have post-CT-TNB hemoptysis, and the orange line represents the assumption that no patients have post-CT-TNB hemoptysis. The decision curve shows that when the threshold probability of a patient is ≥10% (**a**) or between 5 and 90% (**b**), applying this model to predict post-CT-TNB hemoptysis adds more benefit than either the treat-all or the treat-none strategies. Footnotes: *CT-TNB* computed tomography-guided transthoracic needle biopsy

The decision curve for the model and that for the validation is presented in Fig. 5. DCA revealed that when the threshold probability of an individual was ≥10% (in the derivation cohort, Fig. 5a) or between 5 and 90% (in the validation cohort, Fig. 5b), application of this model to predict the risk of post-CT-TNB hemoptysis would add net benefit than applying either the treat-all or treat-none strategies.

## Discussion

In the present study, we developed and validated a risk prediction model for hemoptysis following CT-TNB. This predictive model incorporates five variables: diagnosis of the lesion, lesion characteristics, lesion diameter, procedure time, and puncture distance. The model showed good discrimination predictive ability (AUC: 0.850). We also constructed a nomogram based on these five variables to facilitate the individualized prediction of post-CT-TNB hemoptysis.

In the last few decades, the detection rate of lung lesions has increased, especially in the case of asymptomatic pulmonary nodules and masses [9]. To obtain a pathological diagnosis, several techniques (e.g. CT, endobronchial ultrasound, and virtual bronchoscopic navigation) have been applied for guidance during the biopsy procedure. However, CT-TNB currently remains a frequently used method in clinical practice [6, 10]. Reportedly, the main complications of CT-TNB include hemoptysis, pneumothorax, hemothorax, air embolism, and infection [4, 11, 12]. The incidence of post-CT-TNB hemoptysis, based on different study populations and the type of needle used, varies between 0.5 and 14.4%, and the rate of post-CT-TNB intrapulmonary hemorrhage is 2.9–54.5% [4, 5]. In our study, the incidence of hemoptysis was as high as 42.9%. We speculate that the following factors may have contributed to the high hemoptysis rate. First, we used an 18-gauge coaxial needle rather than fine needle aspiration to perform the biopsies, and it has been reported that the rate of hemoptysis is higher for core biopsies than when fine needle aspiration is used [5]. Second, 96% (418/436) of the patients in our study were subjected to two or more biopsies. Third, 43% (187/436) of the lesions were benign and 39% (172/436) were non-nodular lesions, and more biopsies and larger-gauge needles are recommended when trying to confirm a benign diagnosis [13]. Finally, but most importantly, we defined blood in sputum as hemoptysis in the present study.

There are a number of studies focusing on post-CT-TNB hemoptysis complications [14–16]. However, to our knowledge, no prediction models for CT-TNB-induced hemoptysis have been previously reported. Severe hemoptysis can be life-threatening, and this complication in outpatients would be even more significant. Therefore, it would be helpful for predicting the risk of post-CT-TNB hemoptysis. In this study, we established a risk prediction model based on 5 variables determined by LASSO regression analysis. Regression shrinkage and selection via the LASSO method were first reported by Robert in 1996 [17], and this approach is considered superior to the method of choosing predictors according to the strength of their univariable association with the outcome, especially when there are a large number of variables [18, 19]. All these 5 predictors are easily available clinically. In addition, our prediction model showed both good discrimination ability and calibration.

With regard to its clinical usefulness, we performed DCA to assess whether clinical decisions taken based on this proposed model would improve patient outcomes. DCA, based on different threshold probabilities, could provide insight into the consequences of clinical decisions for clinicians [8, 20, 21]. The DCA based on our model is excellent. It shows that if the threshold probability of an individual was ≥10%, applying the model to predict post-CT-TNB hemoptysis would add a net benefit. Therefore, this model would be useful in the management of post-CT-TNB outpatients. On the one hand, it could reduce patient postoperative anxiety, which induced by post-CT-TNB hemoptysis. On the other hand, it will help physicians screen patients who have a high risk of postoperative hemoptysis, extend their postoperative observation, and provide timely treatment when necessary.

Our study had several major limitations. Firstly, it was a single-center and retrospective evaluation. Although we carried out a validation test of the model, additional independent external verification is warranted to confirm its utility in clinical practice. Secondly, we did not include quantitative measurement of the volume of hemoptysis; therefore, this model can only be used to predict post-CT-TNB hemoptysis, not to distinguish the severity of the hemoptysis. Thirdly, some variables with potential predictive value may help to improve the discrimination ability of the model, such as morphological characteristics of blood vessels, blood flow directions, vessel diameters, local blood pressure and shear stress distribution of all the vessels that involved along the punching pass; however, they were not available in the retrospective data.

## Conclusion

We established a predictive post-CT-TNB hemoptysis model based on 5 predictors. This model not only showed good discrimination ability and calibration characteristics but also demonstrated excellent clinical application potential, as determined by DCA. Therefore, this predictive model may be of great value to facilitate the individualized preoperative prediction of post-CT-TNB hemoptysis.

## Data Availability

The datasets used and/or analyzed during the current study are available from the corresponding author on reasonable request.

## Abbreviations

CT-TNB: Computed tomography-guided transthoracic needle biopsy
COPD: Chronic obstructive pulmonary disease
LASSO: Least absolute shrinkage and selection operator
AUROC: Area under the receiver operating characteristic curve
DCA: Decision curve analysis
CI: Confidence interval
